# The Concordance of Care for Age Related Macular Degeneration with the Chronic Care Model: A Multi-Centered Cross-Sectional Study

**DOI:** 10.1371/journal.pone.0108536

**Published:** 2014-10-07

**Authors:** Stefan Markun, Elisabeth Brändle, Avraham Dishy, Thomas Rosemann, Anja Frei

**Affiliations:** 1 Institute of General Practice and Health Service Research, University of Zurich, University Hospital of Zurich, Zurich, Switzerland; 2 Department of Ophthalmology, Cantonal Hospital Aarau, Aarau, Switzerland; 3 Institute of Social and Preventive Medicine, University of Zurich, Zurich, Switzerland; Canadian Agency for Drugs and Technologies in Health, Canada

## Abstract

**Aims:**

The aim of the study was to assess the concordance of care for age related macular degeneration with the evidence-based framework for care for chronic medical conditions known as the chronic care model. Furthermore we aimed to identify factors associated with the concordance of care with the chronic care model.

**Methods:**

Multi-centered cross-sectional study. 169 patients beginning medical treatment for age related macular degeneration were recruited and analyzed. Patients completed the Patient Assessment of Chronic Illness Care (PACIC) questionnaire, reflecting accordance to the chronic care model from a patient’s perspective, the National Eye Institute Visual Functioning Questionnaire-25 (NEI-VFQ-25) and Patient Health Questionnaire (PHQ-9). Visual acuity and chronic medical conditions were assessed. Nonparametric tests and correlation analyses were performed, also multivariable regression analysis.

**Results:**

The median PACIC summary score was 2.4 (interquartile range 1.75 to 3.25), the lowest PACIC subscale score was “follow-up/coordination” with a median of 1.8 (interquartile range 1.00 to 2.60). In multivariable regression analysis the presence of diabetes type 2 was strongly associated with low PACIC scores (coefficient = −0.85, p = 0.007).

**Conclusion:**

Generally, care for patients with age related macular degeneration by ophthalmologists is in moderate concordance with the chronic care model. Concerning follow-up and coordination of health service, large improvements are possible. Future research should answer the question how healthcare delivery can be improved effecting relevant benefits to patients with AMD.

## Introduction

Health care in general is being challenged by the unprecedented increase in chronic conditions. [1], [2] The need to react to this epidemiological transition has led to initiatives, targeting to improve care for chronic conditions. [3] The chronic care model (CCM) developed by Wagner and colleagues is an evidence-based multifaceted recommendation package, targeting to improve care in different categories that are regarded as determinants for adequate care of chronic conditions. [4]–[6] These categories are organization of healthcare, clinical information systems, delivery-system design, decision support, self-management support and community resources. The CCM is supported by the WHO and has gained widespread acceptance because interventions enhancing the concordance with the CCM effectively improved relevant outcomes in chronic diseases such as diabetes, osteoarthritis or depression. [7]–[9] A validated tool to measure the concordance of care with the CCM from the patient’s perspective has been developed [10].

Age related macular degeneration (AMD) is a chronic condition with severe potential impact on diseased patients. In AMD, visual loss is for the greatest part triggered by the growth of abnormal blood vessels in proximity of the retina. These abnormal blood vessels cause leakage of blood constituents with consecutive anatomic disruption, cell death and ultimately loss of central vision. The growth of new blood vessels including the consequences on visual function, however, can be effectively antagonized by therapies targeting the vascular endothelial growth factor A (VEGF-A). [11] These therapies are applied by periodic intravitreal injections. Current treatment strategies aim to administer injections in phases of disease activity notable by sudden deteriorations of visual function. [12], [13] This individualized and patient-centered approach potentially reduces unnecessary injections, however, it requires health services to provide a thorough follow-up management. Furthermore patients need to be adequately informed and empowered as they take great responsibility by self-monitoring their disease. This is especially important in stages of presumed disease inactivity, when re-occurrence of neovascularizations should prompt immediate re-uptake of an anti-VEGF-A therapy capable of slowing down or even avert permanent visual loss. Such features of healthcare delivery are represented in the CCM, which is therefore relevant in AMD on both the caregivers’ and the patients’ levels. Whether care for AMD is in concordance with the CCM, however, is currently unknown.

The aim of this study was to assess if and to what extent care for patients with AMD is in concordance with the CCM in Swiss ophthalmology clinics (1). Furthermore we aimed to identify factors that determine the concordance of care with the CCM (2).

## Materials and Methods

### Study design

This is a cross-sectional study, based on the baseline data gathered for the randomized trial “The chronic care for age-related macular degeneration study” (CHARMED). The study has been registered at Current Controlled Trials (ISRCTN32507927), the study protocol is publically available. [14] In brief about 20 ophthalmologists from 20 leading ophthalmology clinics in Switzerland providing therapy for patients with AMD were invited to participate in the study by a formal letter. Participating ophthalmologists where trained to gather outcome measures in a standardized format. Also in every participating clinic chronic care coaches where trained to deliver chronic care and conduct structured interviews for outcome measurements. According to the trial’s power calculation 352 patients where intended to be recruited, however, recruiting was stopped after 20 months when the number of newly recruited patients per month reached zero.

### Ethics statement

Ethics committee approval was obtained (ethics committee of the Canton Zurich, KEK-ZH-NR: 2010-04391/1). The study was performed adhering to the tenets of the Declaration of Helsinki and according to Good Clinical Practice Guidelines.

### Patients

Patients were recruited consecutively from April 1 2011 until January 28 2013 by the participating ophthalmologists during clinical visits.

To be eligible for inclusion, patients were required to meet the following inclusion criteria: Diagnosis of wet AMD, age above 50 years, eligible for antiangiogenic drug therapy and visual acuity of at least 20 letters in the assessment with the Early Treatment Diabetic Retinopathy Study (ETDRS) chart. Written informed consent in the study participation was obtained before any study related procedures were taken. Exclusion criteria were former invasive medical treatment for AMD, severe general illness (i.e. advanced malignant tumors or dementia), severe psychological illness and insufficient German or French language skills.

### Measures and data collection

Data collection was performed directly after written informed consent was obtained. A questionnaire was filled in by the ophthalmologists containing the following measures retrieved at the recruitment visit: Visual acuity (using ETDRS charts, standardized measurements were assured by conducting a visit with a teaching session at each ophthalmology clinic); central retinal thickness by optical coherence tomography; specific comorbidities such as hypertension, diabetes type 2, diabetic retinopathy, coronary artery disease, congestive heart failure, stroke or transient ischemic attack, asthma or chronic obstructive lung disease and neoplasms; family anamnesis of wet AMD; smoking status; current medication; estimation of the patients compliance on a four-point scale (range from 1 = very good to 4 = very bad).

A second questionnaire was given to the patients at the recruiting visit to fill in and return directly to the University of Zurich using a readily stamped and accordingly addressed envelope. The questionnaire was self-administered and contained the Patient Assessment of Chronic Illness Care (PACIC) as a measure for concordance of care with the CCM, [10] the PHQ-9 questionnaire as a measure for depression [15] and questions about socio-demographic data and health service utilization.

The PACIC is a validated self-administered instrument that measures the concordance of care with the CCM from the patient’s perspective. According to the key-elements of the CCM the PACIC is organized containing five essential categories of chronic care asked in 20 individual items. In specific these categories are patient activation, delivery system design/decision support, goal setting/tailoring, problem solving/contextual counselling and follow-up/coordination. Each of the individual items are scored on a five point Likert-scale ranging from “almost never” ( = 1, corresponding to poorest concordance with the CCM) to “almost always” ( = 5, corresponding to highest concordance with the CCM). The PACIC summary score is the mean score of the 20 individual items and gives an overall rating of the concordance with the CCM. Five subscales are defined that allow estimations of the CCM concordance with the respective essential categories of chronic care. Such as the CCM, the PACIC itself has shown to be associated with favorable outcomes in chronic conditions. [16] The PACIC summary score was our predefined primary outcome.

The Patient Health Questionnaire (PHQ-9) is a self-administered tool that allows rapid screening for depression and rating severity that recently has showed consistency in patients with visual impairment. [17], [18] The PHQ-9 was introduced in the measurements as a confounder control, because depression is known to be highly prevalent among patients with AMD and might influence outcomes of the CHARMED randomized trial (not discussed in this article) [19].

In a face-to-face or telephone interview with the patients the trained chronic care coaches operated the National Eye Institute Visual Functioning Questionnaire-25 (NEI-VFQ-25) in the interviewer administered format. [20] The NEI-VFQ-25 is a validated measure of the visual disability specific quality of life and functioning, it consists of 25 vision-targeted questions that generate 11 subscales of vision related health and functioning [21], [22].

Patients and ophthalmologists’ answers remained concealed from each other; the University of Zurich had no access to the patients’ personal informations ensuring anonymity.

### Statistical analysis

Continuous variables are presented as means and standard deviations or medians and interquartile ranges if not otherwise declared; categorical data is presented as frequencies and percentages. Bivariate association between the PACIC summary score and continuous variables were conducted using Spearman correlations, between PACIC summary score and categorical variables using Mann-Whitney-U Test and Kruskal-Wallis Test (more than two groups). Potential determinants of the PACIC summary score were investigated by multivariable regression model. We included all variables in the model that showed both a significant bivariate relationship with the PACIC summary score on a 5% level and could be interpreted in terms of content as potential determinants. We further controlled for the cluster-effect of clinics, thus taking into account that patient observations are not independent, i.e. observations in one cluster tend to be more similar to each other than to individuals in the rest of the sample. A two-sided alpha of 0.05 was set as level of significance for all comparisons. Missing data were left as missing, for the construction of the PACIC summary score one missing item (out of 20) was allowed. Analyses were calculated using the software SPSS version 21.0 and STATA version 12.

## Results

### Ophthalmologist characteristics

Twelve different ophthalmologists from twelve different clinics could be recruited for the study. Amongst the clinics, three different categories of organization type were found: Three clinics were single handed practices (median number of patients recruited = 7, range 2–12), five clinics were group practices (median number of patients recruited = 5, range 1–18), four were clinics run within hospitals (median number of patients recruited = 25, range 11–54).

### Patient characteristics and clinical measures

In total, 169 patients were enrolled in the study. 21 (12.4%) patients were recruited in single handed practices, 32 (18.9%) in group practices and 116 (68.6%) in clinics run within hospitals. 107 (63.3%) of the patients were female, the mean age was 76.7 (±8.0) years. The average of total education years absolved was 12.0 (±3.4). In all patients, treatment with ranibizumab was started. Further characteristics are shown in Table 1.

**Table 1:** Demographical and social characteristics of study patients, smoking status and patient compliance from the ophthalmologist’s perspective; table n = 169.

*Variable*	*Category*	*n*	*percent*
Gender	Male	62	36.7
	Female	107	63.3
	Missing information	0	0.0
Age	<60 years old	3	1.8
	60–69 years old	33	19.5
	70–79 years old	69	40.8
	80–89 years old	58	34.3
	≥90 years old	6	3.6
	Missing information	0	0.0
Living situation	Living with partner or family	103	60.9
	Living alone	53	31.4
	Missing information	13	7.7
Working situation	Still working	12	7.1
	Retired	144	85.2
	Missing information	13	7.7
Education years completed	≤6 years	2	1.2
	7 to 9 years	41	24.3
	10 to 12 years	56	33.1
	≥13 years	56	33.1
	Missing information	14	8.3
Smoking status	Current smoker	26	15.4
	Former smoker	49	29.0
	Never smoker	94	55.6
	Missing information	0	0.0
Compliance	Very good	96	56.8
	Rather good	69	40.8
	Rather bad	3	1.8
	Very bad	1	0.6
	Missing information	0	0

The mean number (± standard deviation) of correctly identified ETDRS letters was 74.1 (±14.9) with the better eye and 52.6 (±18.9) with the worse eye. The mean (± standard deviation) NEI-VFQ-25 composite score was 83.8 (±12.4). The mean general health rating (not component of the composite score) was 54.6 (±19.5). Detailed vision-specific information is given in Table 2.

**Table 2:** Vision-specific variables of study patients: visual acuity was assessed with the Early Treatment Diabetic Retinopathy Study (ETDRS) Chart; disability was assessed with the National Eye Institute Visual Functioning Questionnaire-25 (NEI-VFQ-25), range 0 to 100 (0 represents worst, 100 represents best possible visual functioning); table n = 169.

*Variable*	*Category*	*Mean*	*Standard deviation*	*n*	*Percent*
ETDRS Visual acuity better eye	Total ETDRS letters correct	74.1	14.9		
	<31 ETRDS letters correct			4	2.4
	31–50 ETRDS letters correct			9	5.3
	51–70 ETRDS letters correct			30	17.8
	>70 ETRDS letters correct			118	69.8
	Missing information			8	4.7
ETDRS Visual acuity worse eye	Total ETDRS letters correct	52.6	18.9		
	<31 ETRDS letters correct			18	10.7
	31–50 ETRDS letters correct			48	28.4
	51–70 ETRDS letters correct			68	40.2
	>70 ETRDS letters correct			27	16.0
	Missing information			8	4.7
NEI-VFQ-25 subscales*	General health	54.6	19.5		
	General vision	67.0	13.6		
	Ocular pain	90.0	15.8		
	Near activities	76.4	20.6		
	Distant activities	79.9	19.9		
	Social functioning	94.0	14.2		
	Mental health	78.7	18.7		
	Role difficulties	82.1	24.6		
	Dependency	93.8	17.0		
	Driving	68.8	33.9		
	Color vision	96.2	13.8		
	Peripheral vision	88.7	18.9		

*) Missing information for all items ranged from 6 to 8, except for “Driving” which was unanswered in 83 cases.

In 111 (65.7%) of the patients at least one co-occurring chronic medical condition was present. 99 (58.6%) of the patients had cardiovascular comorbidities. The median number of ophthalmologist consultations within one year was 2 (interquartile range 1 to 3), the median number of GP consultations was 3 (interquartile range 1 to 6). 37 (21.9%) patients had at least one hospitalization during the last year, 27 (16.0%) had emergency hospitalizations. Data about comorbidities and healthcare utilization is displayed in Table 3.

**Table 3:** Comorbidity and Health Service Utilization; table n = 169.

*Variable*	*Category*	*n*	*percent*
Number of comorbidities	0 comorbidities	58	34.3
	1 comorbidity	60	35.5
	2 comorbidities	34	20.1
	3 comorbidities	10	5.9
	≥4 comorbidities	6	3.6
	Missing information	1	0.6
Specific comorbidities	Hypertension	92	54.4
	Diabetes type 2	14	8.3
	Diabetic retinopathy	0	0.0
	Coronary artery disease	29	17.2
	Congestive heart failure	8	4.7
	Stroke/TIA1)	14	8.3
	Asthma/COPD2)	16	9.5
	Neoplasm	11	6.5
Depression according to PHQ-93)	No depression	116	68.6
	Mild depression	32	18.9
	Moderate major depression	3	1.8
	Severe major depression	2	1.2
	Missing information	16	9.5
Number of ophthalmologist consultations last year	1–2 consultations	87	51.5
	3–4 consultations	38	22.5
	5–6 consultations	14	8.3
	≥7 consultations	8	4.7
	Missing information	22	13.0
Number of GP4) consultations last year	0–2 consultations	65	38.5
	3–4 consultations	36	21.3
	5–6 consultations	23	13.6
	≥7 consultations	31	18.3
	Missing information	14	8.3
Number of days in hospital last year	0 days	117	69.2
	1–3 days	8	4.7
	4–10 days	18	10.7
	≥11 days	11	6.5
	Missing information	15	8.9

1) TIA = Transient ischemic attack;

2) COPD = Chronic obstructive pulmonary disease;

3) PHQ-9 = Patient Health Questionnaire-9;

4) GP = General practitioner.

### PACIC scores

**Figure 1:**
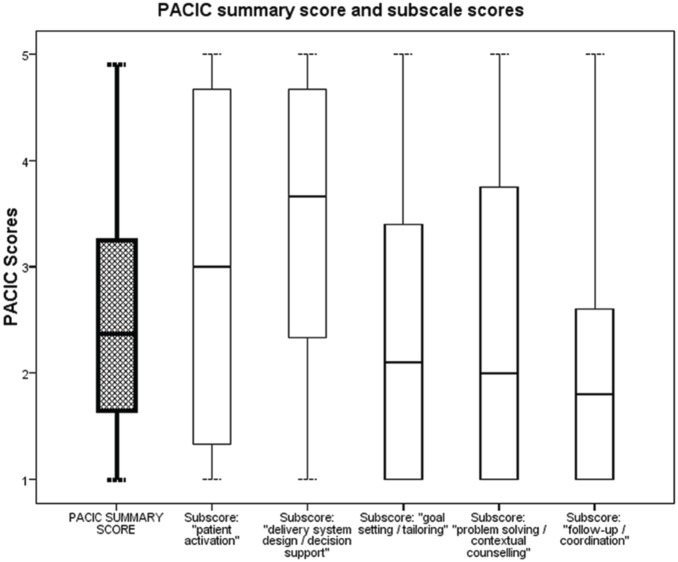
Distribution of PACIC summary score and subscale scores. PACIC scores are represented in boxplots. The PACIC summary score is displayed on the left, other boxplots are the respective PACIC subscale scores. The lower and the upper margin of the box indicate the 25^th^ and 75^th^ percentile respectively. The bar inside the box indicates the median. Whiskers extend to the most extreme data point within 1.5 times the interquartile range.

The median PACIC summary score was 2.4 (interquartile range 1.75 to 3.25). There were substantial differences between the different PACIC subscale scores. The subscale score “follow-up/coordination” resulted lowest with a median subscale score of 1.8 (interquartile range 1.00 to 2.60), corresponding with an average rating of the items in the PACIC questionnaire somewhat lower than “generally not”. The highest subscale score was “delivery system design/decision support” with a median subscale score of 3.7 (interquartile range 2.33 to 4.67), corresponding with an average rating of the items in the PACIC questionnaire between “sometimes” and “most of the time”. Details about the distribution of the PACIC summary score and subscale results are displayed in Table 4 and Figure 1.

**Table 4:** Patient Assessment of Chronic Illness Care (PACIC) scores; range 1 to 5, 1 corresponds to poorest concordance with the Chronic Care Model (CCM), 5 corresponds to highest concordance with the CCM; for the construction of the PACIC summary score one missing item was allowed.

*PACIC Scores*	*Valid n*	*Median*	*Interquartile range*
Summary score1)	131	2.4	1.75 to 3.25
Subscale “Patient activation”	141	3.0	1.67 to 4.67
Subscale “Delivery systemdesign/decision support”	133	3.7	2.33 to 4.67
Subscale “Goal setting/tailoring”	138	2.2	1.30 to 3.40
Subscale “Problem solving/contextualcounselling”	138	2.0	1.19 to 3.81
Subscale “Follow-up/coordination”	139	1.8	1.00 to 2.60

1) For calculation of PACIC summary score one missing item (out of twenty) in the questionnaire was allowed.

### Bivariate associations with PACIC summary score

The PACIC summary score was not significantly associated with the practice organization type (single handed, group practices and ambulatory clinics in hospitals: independent sample Kruskal-Wallis Test p = 0.187). No significant associations with the PACIC summary score were found for patients’ socio-demographic characteristics, smoking status or physician-reported compliance. Also no significant association with the PACIC summary score was found for the number of comorbidities and for the PHQ-9. The presence of diabetes type 2 was significantly associated with lower PACIC scores (median [interquartile range] PACIC summary score of group with diabetes type 2 = 1.5 [1.11 to 2.13]), group without diabetes type 2 = 2.7 [1.83 to 3.28]; Mann Whitney U Test p = 0.002). Also the presence of coronary artery disease was significantly associated with lower PACIC scores (median [interquartile range] PACIC summary score of group with coronary artery disease = 2.0 [1.37 to 2.89]), group without coronary artery disease = 2.7 [1.80 to 3.30]; Mann Whitney U Test p = 0.037). No vision-specific variable showed association with the PACIC summary score.

### Multivariate associations with PACIC summary score

For the multivariable regression model we considered the comorbidity diabetes type 2 and coronary artery disease (both significant in bivariate analysis), gender and age (potential confounders) and visual acuity of the better eye (as an indicator for the severity of visual loss). The model was controlled for the cluster-effect originating from the different study clinics.

After application of the model, the only significant determinant of the PACIC summary score was diabetes type 2 (coefficient = −0.85, p = 0.007). Coronary artery disease closely missed significance (coefficient = −0.44, p = 0.059), the other variables showed no association (Table 5).

**Table 5:** Adjusted Regression Coefficients (95%CI) for the Patient Assessment of Chronic Illness Care (PACIC) summary score (n = 126)1).

*Variable*	*Category*	*Adjusted Regression* *Coefficient (95%CI)*
Gender	Male	Ref
	Female	−0.06 (−0.41 to 0.29)
Age	Years	0.01 (−0.02 to 0.03)
Visual acuity of better eye	ETDRS	0.008 (−0.004 to 0.02)
Diabetes Type 2	Absent	Ref
	Present	−0.85 (−1.47 to −0.24)*
Coronary artery disease	Absent	Ref
	Present	−0.44 (−0.91; 0.02)†

1) Multivariable regression model adjusted for all determinants in column and additionally controlled for the cluster-effect originating from the different study clinics (n = 126 patients with complete determinant and PACIC data); there was a significant cluster-effect resulting in an intra-class correlation coefficient (ICC) of 9.1% (p = 0.027).

*p = 0.007.

† p = 0.059.

## Discussion

The CCM is an evidence-based template for the care for patients with chronic illnesses. We aimed to measure the concordance of care with the CCM in patients treated for AMD. We found that patients perceived moderate overall concordance with the CCM, especially low concordance was found in the “follow-up/coordination” subscale score.

A broad spectrum of different types of ophthalmology clinics participated in the study. Interestingly the PACIC summary score showed no significant association with the organization type of the clinics. Although relevant structural differences between hospital-run ophthalmology clinics and single handed ophthalmology practices must be assumed, those differences did not affect the concordance with the CCM from the patients perspective.

According to the inclusion and exclusion criteria, patients that just newly qualified for antiangiogenic therapy were recruited in this study. The sample is thus representative for AMD patients at the beginning of their chronic care situation. The relatively intact ETDRS visual acuity and the high NEI-VFQ-25 scores showed that these patients are mostly in early stages of the disease and have therefore the highest potential to benefit from preventive interventions enabled by successful healthcare delivery.

PACIC scores we identified, however, were modest. The subscale score “follow-up/coordination” was particularly low also in comparison to PACIC scores from research done in other chronic conditions such as diabetes, osteoarthritis or inflammatory bowel disease. [23]–[25] In AMD, however, a low concordance of follow-up organization with the CCM is especially undesirable because successful follow-up is regarded to be critical for outcomes. [26] It remains, however, debatable whether the PACIC subscales truly represent the different domains of the CCM, because of the high internal consistency of the total PACIC score itself [10], [27].

Furthermore, we found specific comorbidities to be associated with low PACIC scores in ophthalmological care. In the case of diabetes type 2 this is troublesome because patients with both AMD and diabetes are in the highest need for successful chronic care as their visual acuity is endangered by two treatable chronic conditions simultaneously. With our data we cannot explain this finding of co-occurring conditions being associated with decreased PACIC scores. A possible explanation for this finding could be that ophthalmologists feel less responsible for care for multimorbid patients since they assume that the GP coordinates the care for them.

## Limitations

Undoubtedly, the implementation of the different important elements of chronic care is a dynamic process requiring several contacts of healthcare professionals with the individual patient. Patients in our sample, however, were newly entering a chronic care situation for AMD. The PACIC results we obtained might therefore be representative for patients in an early stage of chronic care implementation, thus showing the gaps that still need to be filled. This might especially apply to our results in the “follow-up/coordination” subscale. Only 131 of 169 patients provided enough data to allow calculation of the PACIC summary score. A greater number of cases would have provided more power to detect significant results. Also, we cannot exclude bias from selective answering; we assume, however, that patients dissatisfied with their healthcare would be less motivated to answer the questionnaire, thus causing bias towards false high PACIC scores. PACIC scores in our study would thus tend to be overestimated, the potential for enhancements even greater. Finally, the PACIC score reflects the patient perspective of chronic illness care only, leaving the healthcare provider’s perspective unclear.

## Conclusion

Generally, care for patients with age related macular degeneration by ophthalmologists is in moderate concordance with the chronic care model. Concerning follow-up and coordination of health service, large improvements are possible, for example with follow-up calls by health service professionals (in order to prevent loss of follow-up during inactive stage of the disease) or with coordination of health service between primary care and specialized ophthalmologic care (in order to allocate responsibilities and avoid under- or overtreatment especially if additional chronic diseases as diabetes exist). Future research should answer the question how healthcare delivery can be improved effecting relevant benefits to patients with AMD.

## References

[1] Lochner KA, Cox CS (2013) Prevalence of multiple chronic conditions among Medicare beneficiaries, United States, 2010. Prev Chronic Dis 10: E61. 23618541 10.5888/pcd10.120137 PMC3652723

[2] Thorpe KE, Ogden LL, Galactionova K (2010) Chronic conditions account for rise in Medicare spending from 1987 to 2006. Health Aff (Millwood) 29: 718–724. 20167626 10.1377/hlthaff.2009.0474

[3] Tsai AC, Morton SC, Mangione CM, Keeler EB (2005) A meta-analysis of interventions to improve care for chronic illnesses. Am J Manag Care 11: 478–488. 16095434 PMC3244301

[4] Wagner EH (1998) Chronic disease management: what will it take to improve care for chronic illness? Eff Clin Pract 1: 2–4. 10345255

[5] Wagner EH, Austin BT, Davis C, Hindmarsh M, Schaefer J, et al (2001) Improving chronic illness care: translating evidence into action. Health Aff (Millwood) 20: 64–78. 11816692 10.1377/hlthaff.20.6.64

[6] Epping-Jordan JE, Pruitt SD, Bengoa R, Wagner EH (2004) Improving the quality of health care for chronic conditions. Qual Saf Health Care 13: 299–305. 15289634 10.1136/qshc.2004.010744 PMC1743863

[7] Stellefson M, Dipnarine K, Stopka C (2013) The chronic care model and diabetes management in US primary care settings: a systematic review. Prev Chronic Dis 10: E26. 23428085 10.5888/pcd10.120180 PMC3604796

[8] Rosemann T, Joos S, Laux G, Gensichen J, Szecsenyi J (2007) Case management of arthritis patients in primary care: a cluster-randomized controlled trial. Arthritis Rheum 57: 1390–1397. 18050178 10.1002/art.23080

[9] Gensichen J, von Korff M, Peitz M, Muth C, Beyer M, et al (2009) Case management for depression by health care assistants in small primary care practices: a cluster randomized trial. Ann Intern Med 151: 369–378. 19755362 10.7326/0003-4819-151-6-200909150-00001

[10] Glasgow RE, Wagner EH, Schaefer J, Mahoney LD, Reid RJ, et al (2005) Development and validation of the Patient Assessment of Chronic Illness Care (PACIC). Med Care 43: 436–444. 15838407 10.1097/01.mlr.0000160375.47920.8c

[11] Vedula SS, Krzystolik MG (2008) Antiangiogenic therapy with anti-vascular endothelial growth factor modalities for neovascular age-related macular degeneration. Cochrane Database Syst Rev: CD005139. 10.1002/14651858.CD005139.pub2 PMC4267250 18425911

[12] Chakravarthy U, Williams M (2013) Group AG (2013) The Royal College of Ophthalmologists Guidelines on AMD: Executive Summary. Eye (Lond) 27: 1429–1431. 24158023 10.1038/eye.2013.233 PMC3869519

[13] Brand CS (2012) Management of retinal vascular diseases: a patient-centric approach. Eye (Lond) 26 Suppl 2 S1–16. 22495396 10.1038/eye.2012.32 PMC3335302

[14] Frei A, Woitzek K, Wang M, Held U, Rosemann T (2011) The chronic care for age-related macular degeneration study (CHARMED): study protocol for a randomized controlled trial. Trials 12: 221. 21985296 10.1186/1745-6215-12-221 PMC3214138

[15] Lowe B, Kroenke K, Herzog W, Grafe K (2004) Measuring depression outcome with a brief self-report instrument: sensitivity to change of the Patient Health Questionnaire (PHQ-9). J Affect Disord 81: 61–66. 15183601 10.1016/S0165-0327(03)00198-8

[16] Schmittdiel J, Mosen DM, Glasgow RE, Hibbard J, Remmers C, et al (2008) Patient Assessment of Chronic Illness Care (PACIC) and improved patient-centered outcomes for chronic conditions. J Gen Intern Med 23: 77–80. 18030539 10.1007/s11606-007-0452-5 PMC2173922

[17] Lamoureux EL, Tee HW, Pesudovs K, Pallant JF, Keeffe JE, et al (2009) Can clinicians use the PHQ-9 to assess depression in people with vision loss? Optom Vis Sci 86: 139–145. 19156007 10.1097/OPX.0b013e318194eb47

[18] Kroenke K, Spitzer RL, Williams JB (2001) The PHQ-9: validity of a brief depression severity measure. J Gen Intern Med 16: 606–613. 11556941 10.1046/j.1525-1497.2001.016009606.x PMC1495268

[19] Brody BL, Gamst AC, Williams RA, Smith AR, Lau PW, et al (2001) Depression, visual acuity, comorbidity, and disability associated with age-related macular degeneration. Ophthalmology 108: 1893–1900 discussion 1900–1891. 11581068 10.1016/s0161-6420(01)00754-0

[20] Mangione CM, Lee PP, Gutierrez PR, Spritzer K, Berry S, et al (2001) Development of the 25-item National Eye Institute Visual Function Questionnaire. Arch Ophthalmol 119: 1050–1058. 11448327 10.1001/archopht.119.7.1050

[21] Revicki DA, Rentz AM, Harnam N, Thomas VS, Lanzetta P (2010) Reliability and validity of the National Eye Institute Visual Function Questionnaire-25 in patients with age-related macular degeneration. Invest Ophthalmol Vis Sci 51: 712–717. 19797233 10.1167/iovs.09-3766

[22] Mangione CM (2000) The National Eye Institute 25-Item Visual Function Questionnaire (VFQ-25) - Version 2000. Available: http://www.nei.nih.gov/resources/visionfunction/manual_cm2000.pdf. Accessed 2013 Nov 6.

[23] Rosemann T, Laux G, Droesemeyer S, Gensichen J, Szecsenyi J (2007) Evaluation of a culturally adapted German version of the Patient Assessment of Chronic Illness Care (PACIC 5A) questionnaire in a sample of osteoarthritis patients. J Eval Clin Pract 13: 806–813. 17824876 10.1111/j.1365-2753.2007.00786.x

[24] Frei A, Herzog S, Woitzek K, Held U, Senn O, et al (2012) Characteristics of poorly controlled Type 2 diabetes patients in Swiss primary care. Cardiovasc Diabetol 11: 70. 22704274 10.1186/1475-2840-11-70 PMC3403845

[25] Randell RL, Long MD, Martin CF, Sandler RS, Chen W, et al (2013) Patient perception of chronic illness care in a large inflammatory bowel disease cohort. Inflammatory bowel diseases 19: 1428–1433. 23574758 10.1097/MIB.0b013e3182813434 PMC4600129

[26] Rasmussen A, Bloch SB, Fuchs J, Hansen LH, Larsen M, et al (2013) A 4-Year Longitudinal Study of 555 Patients Treated with Ranibizumab for Neovascular Age-related Macular Degeneration. Ophthalmology 120: 2630–2636. 23830760 10.1016/j.ophtha.2013.05.018

[27] Gugiu C, Coryn CL, Applegate B (2010) Structure and measurement properties of the Patient Assessment of Chronic Illness Care instrument. J Eval Clin Pract 16: 509–516. 20210824 10.1111/j.1365-2753.2009.01151.x

